# Vibration stimulation enhances robustness in teleoperation robot system with EEG and eye-tracking hybrid control

**DOI:** 10.3389/fbioe.2025.1591316

**Published:** 2025-05-08

**Authors:** Wenbin Zhang, Tianjie Wang, Chaolong Qin, Baoguo Xu, Hexuan Hu, Tong Wang, Ying Shen

**Affiliations:** ^1^ The College of Computer Science and Software Engineering, Hohai University, Nanjing, China; ^2^ Department of Rehabilitation Medicine, The First Affiliated Hospital of Nanjing Medical University, Nanjing, China; ^3^ The State Key Laboratory of Digital Medical Engineering, School of Instrument Science and Engineering, Southeast University, Nanjing, China

**Keywords:** brain-computer interface, vibrotactile stimulation, motor imagery, teleoperation robot, eye tracking

## Abstract

**Introduction:**

The application of non-invasive brain-computer interfaces (BCIs) in robotic control is limited by insufficient signal quality and decoding capabilities. Enhancing the robustness of BCIs without increasing the cognitive load remains a major challenge in brain-control technology.

**Methods:**

This study presents a teleoperation robotic system based on hybrid control of electroencephalography (EEG) and eye movement signals, and utilizes vibration stimulation to assist motor imagery (MI) training and enhance control signals. A control experiment involving eight subjects was conducted to validate the enhancement effect of this tactile stimulation technique.

**Results:**

Experimental results showed that during the MI training phase, the addition of vibration stimulation improved the brain region activation response speed in the tactile group, enhanced the activation of the contralateral motor areas during imagery of non-dominant hand movements, and demonstrated better separability (p = 0.017). In the robotic motion control phase, eye movement-guided vibration stimulation effectively improved the accuracy of online decoding of MI and enhanced the robustness of the control system and success rate of the grasping task.

**Discussion:**

The vibration stimulation technique proposed in this study can effectively improve the training efficiency and online decoding rate of MI, helping users enhance their control efficiency while focusing on control tasks. This tactile enhancement technology has potential applications in robot-assisted elderly care, rehabilitation training, and other robotic control scenarios.

## 1 Introduction

BCI is a type of human-computer interaction system that enables direct interaction between the brain and the external environment or equipment without relying on the peripheral nerve and muscle systems (Vidal, 1973). Based on signal acquisition techniques, BCIs are primarily divided into invasive and non-invasive BCIs. Non-invasive BCIs use electrodes placed on the scalp to acquire EEG signals, offering advantages in terms of safety, convenience, and wide-ranging applications. Common non-invasive BCI paradigms include MI, Steady-state Visual Evoked Potentials (SSVEP), and Auditory Evoked Potentials (AEP) (Hajcak et al., 2019; Norcia et al., 2015). Among these, MI is the most widely used spontaneous EEG paradigm, with broad applications in fields such as rehabilitation and robotic control (McFarland and Wolpaw, 2017; Chaudhary et al., 2016; Lian et al., 2024).

The use of BCI to realize the robust control of robots has always been a popular research direction in the academic field. At present, invasive BCI systems can control various functional tasks of robots, such as handshaking and target tracking (Collinger et al., 2013; Hochberg et al., 2012; Handelman et al., 2022). Non-invasive BCI systems are limited by poor signal quality and spatial resolution. They mainly employed passive elicitation paradigms, such as SSVEP (Yang et al., 2017; Chen et al., 2018) and P300 (Pathirage et al., 2013; Hu et al., 2024), to control robots. However, the drawback of these paradigms is that the control signals are induced by external stimuli, which may make the online control method less natural and result in poor user experience. Simultaneously, the active BCI paradigm based on sensorimotor rhythm has also been applied to the control of robotic arms (Iáñez et al., 2010; Savić et al., 2023). Owing to the low signal-to-noise ratio of EEG signals and the limited number of instructions and decoding accuracy, it is difficult for such paradigms to decode continuous motion with a high information transmission rate and cannot smoothly control robotic arms in three-dimensional space (Kim et al., 2015).

As a safe, low-cost, wearable, and highly acceptable tactile stimulation method, vibration stimulation can influence the somatosensory cortex by activating mechanoreceptors in the skin (Klatzky, 2024). Previous studies have used tactile stimulation to enhance the performance of the MI paradigm by closing the sensory-motor loop (Yao et al., 2013; Zhang et al., 2022; 2021). To achieve robust MI control, long-term training is always required to improve decoding accuracy. Yao et al. applied vibration stimulation to the ipsilateral hand during MI tasks, providing tactile feedback to enhance neural feedback training efficiency (Zhong et al., 2022). Ming et al. used a hybrid BCI paradigm combining tactile stimulation and MI (Yi et al., 2017). They integrated Event-related Synchronization/Desynchronization (ERS/ERD) with steady-state somatosensory evoked potentials (SSSEP) to improve the decoding rate of the hybrid paradigm by approximately 14 % compared to the pure MI paradigm. However, most related studies have applied vibration stimulation in an open-loop manner, and because the MI paradigm is spontaneous, it is difficult to apply stimulation only to the imagined side. Additionally, applying bilateral tactile stimulation is believed to not improve the overall performance of the left-right hand MI paradigm.

In current brain-controlled robot systems, tactile stimulation is mainly used to provide feedback on control results or to increase the number of commands. Peters et al. used a robotic arm to simulate limb movements and enhance the decoding rate of the MI paradigm by providing kinesthetic feedback (Gomez-Rodriguez et al., 2011a). Kim et al. developed a wheelchair control system based on SSSEP, where users controlled the wheelchairâ€™s left-right turning and forward motion through selective vibration feedback provided by actuators placed on the index fingers and toe (Kim et al., 2016). This method achieved higher success rates and shorter times for obstacle avoidance tasks than traditional MI control. However, such effective tactile stimulation often requires large equipment or complex pre-calibration procedures, making it challenging for large-scale applications (Zeng et al., 2024; Gomez-Rodriguez et al., 2011b). Therefore, the natural and efficient integration of tactile stimulation into robot control systems to enhance the overall performance of MI-BCI without increasing the cognitive load is an important research issue.

The current mainstream control strategies for brain-controlled systems mainly include process control and goal selection. In process control, users can set the robotâ€™s detailed motion parameters (e.g., direction, speed, and distance) using the BCI. This control strategy is highly flexible, allowing users to freely control the robot movements. However, even with invasive BCIs, achieving dexterous control of robotic arms entirely through EEG signals is challenging (Meng et al., 2016). The output of BCIs may not be sufficiently reliable, and the limited number of commands makes it difficult to complete fine-control tasks. In contrast, goal selection control is relatively simpler, as users only need to select specific task options without worrying about the detailed operation process. However, this reduces the control authority of the user, preventing them from intervening in the control process, which can lead to frustration. Therefore, more research is focused on combining both strategies, using technologies such as machine vision to merge user control with autonomous robot control, forming a shared control strategy that allows users to participate as much as possible in the control process, while the robot autonomously handles fine and accurate movement. Hybrid BCIs that combine machine vision, autonomous positioning, and other technologies have helped paralyzed patients complete object grasping and moving tasks. However, studies have shown that users do not expect all control tasks to be performed automatically by assistive devices (Kim et al., 2011). Therefore, it is crucial to improve user participation through tactile stimulation to make the control interaction process more natural in brain-controlled robotic systems.

To this end, this study designs a teleoperation robot system based on the hybrid control of MI-BCI and eye movement signals. The eye gaze signal compensates for the insufficient number of MI commands, while vibration stimulation is naturally applied during the control process to close the loop and modulate the activation of the user’s sensorimotor cortex. Eight subjects were recruited for a control experiment to validate the enhancement effect of vibration stimulation on MI training and robot control efficiency. Based on the experimental results, we discusses the integration methods and application prospects of vibration stimulation with the control system.

## 2 Methods

The brain-controlled teleoperation system proposed in this study comprises eight modules. The EEG acquisition module uses an EEG cap and amplifier to collect the subject’s real-time EEG data. The eye movement acquisition module uses an eye tracker to obtain the real-time fixation coordinates of the subject’s gaze on the visual stimulus display screen. The information from these two modules is processed and analyzed by the signal analysis module, which converts it into control commands for the remote robot as well as the control commands for the vibration stimulation. Vibration stimulation module includes two vibration actuators that are controlled according to the commands transmitted by the signal analysis module. The information transmission module uses the TCP/IP protocol to facilitate communication between the remote robot system and the main control system. It is also responsible for transmitting real-time remote images captured by the image acquisition module to the visual display module, which shows the control perspective and graphical buttons on the software interface. Finally, the robot control module uses pre-calibrated position coordinates and control commands sent from the main system to guide the robotic arm to complete the target grasping and placement tasks.

### 2.1 Teleoperation robot system design

**FIGURE 1:**
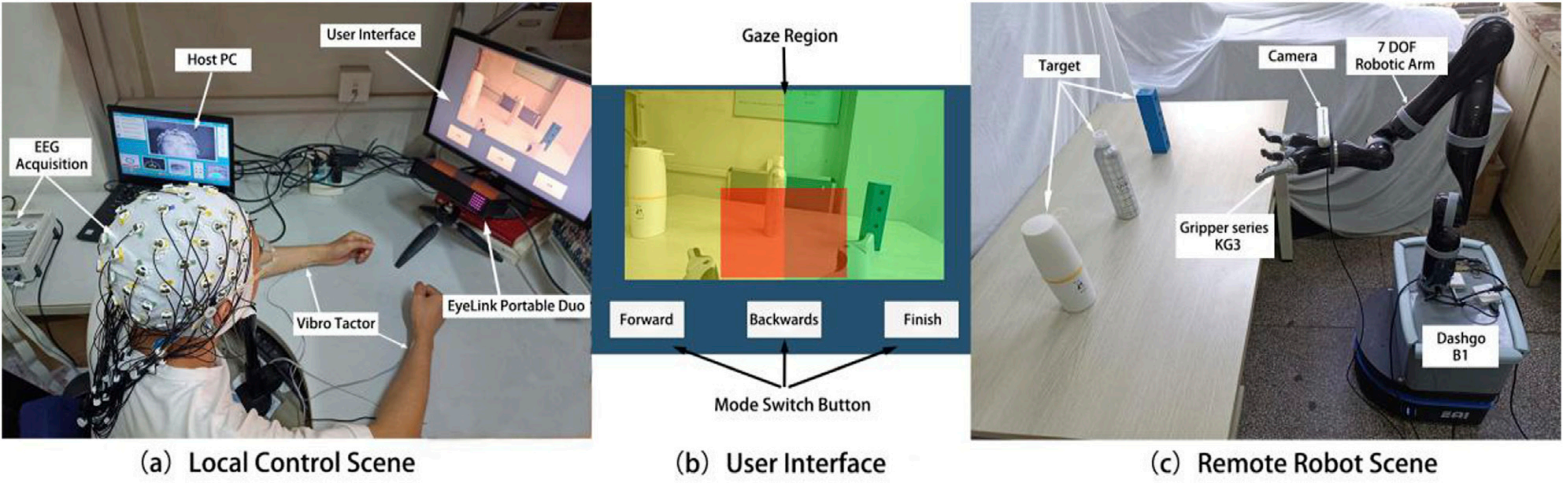
Actual scenes of local control **(a)**, visual control interface **(b)**, and remote robot platform **(c)**.

The EEG and eye-gaze based remote control system designed in this study is shown in Figure 1a. The remote control system consisted of an EEG signal acquisition module and an eye movement signal acquisition module. The EEG acquisition module used a BP EEG acquisition device (ActiCAP Systems, BrainProducts GmbH, Germany) to acquire real-time EEG data from 20 channels (FC5, FC1, C3, CP5, CP1, CP6, CP2, Cz, C4, FC6, FC2, FC3, C1, C5, CP3, CPz, CP4, C6, C2, and FC4)Â based on the international 10/20 system, which primarily covers sensory-motor-related areas of the frontal and parietal lobes. The sampling frequency was 1,000 Hz and the electrode impedance was maintained below 15 k ω during EEG acquisition. All electrode channels used the FCz channel as the reference and the FPz channel as the ground. The signal was filtered using a 0.1–100 Hz analog bandwidth filter and a 50 Hz notch filter to reduce interference. A portable eye tracking system (Eyelink Portable Duo) was used to track the user’s eye movements and gaze. This eye tracker utilizes pupil and corneal reflections to track eye movement, with a sampling rate of 2000 Hz and an accuracy of 0.15 ° . The eye movement system includes a Host PC that helps the experimenter perform eye movement calibration, adjust thresholds, and record data. Another computer was used to display real-time images and a control interface at the control end. A head support bracket was used to stabilize the user’s head, ensuring that the relative distance between the eyes, eye tracker, and control display remained constant. The gaze fixation signals captured by the eye tracker were the coordinates of the user’s gaze on the control display screen. In this study, gaze fixation within a region for more than 0.5 s was defined as a gaze event.

**FIGURE 2:**
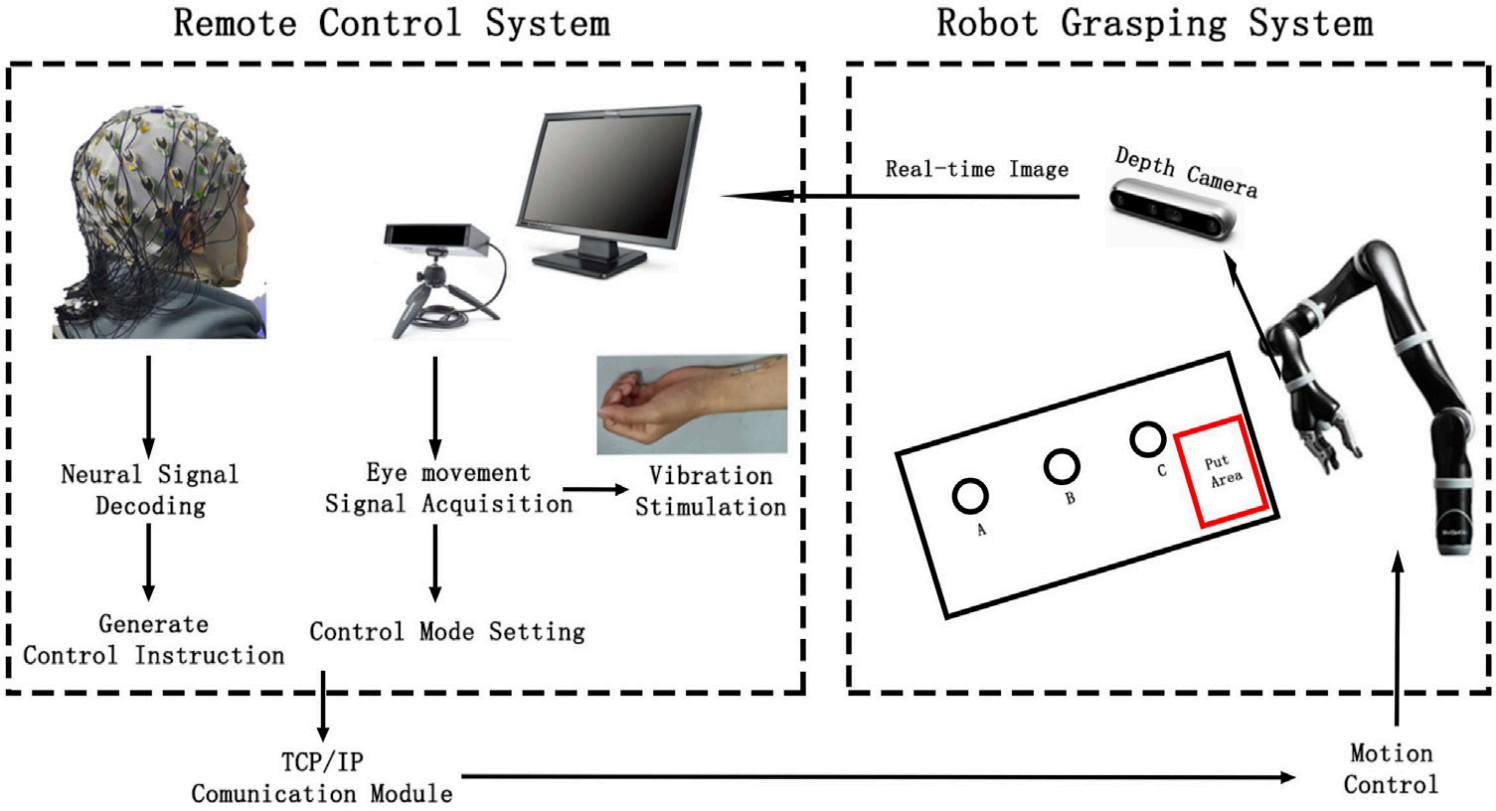
System framework of brain-controlled teleoperation robot.

The EEG signals collected by the EEG system were transmitted in real time to MATLAB software via a wired connection between the control computer and the EEG amplifier. A custom MATLAB program was used to filter and decode the EEG signals online, generating control commands to move the robotic arm during the target selection phase. The real-time gaze position information from the eye movement acquisition system was used to determine the user’s control intention, control the grasping task, and switch the direction and control mode of the robotic arm’s movement. The framework of the entire remote operation robotic grasping system is illustrated in Figure 2. The control commands generated by the remote control system were sent to the robot’s control computer via the TCP/IP protocol. The robot executed control commands based on precalibrated coordinates. After each control command was executed, the end position coordinates were used to determine the relative position for grasping, completing the tasks of grasping, and placing objects A, B, and C automatically.

Vibration stimulation was provided by two piezoelectric actuators (PHAT423535XX, Fyber Labs Inc., Korea). These actuators were fixed on the median nerves of the left and right wrists using medical tape. The vibration frequency was set at 200 Hz, which is within the optimal perception range of the skin for vibration stimulation. This frequency can effectively activate tactile receptors such as Meissner corpuscles. The amplitude was adjusted based on the intensity that was clearly felt by the subjects during the pre-experiment but did not interfere with the MI task. The control interface displayed on the screen, which the subjects gazed at during the control process, is shown in Figure 1b. The interface includes real-time control images and three white rectangles corresponding to the forward, backward, and finish commands. The eye tracker collects the relative position coordinates of the user’s gaze on the control interface, with different regions corresponding to different commands. The colored shading added in Figure 1b helps to intuitively explain the division of gaze regions, where the yellow and green regions correspond to the left and right target areas, respectively, and the red region corresponds to the grasping task determination area.

For the robot platform, a brain-controlled robotic arm (KINOVA Ultra Lightweight Robotic Arm 7 DOF Spherical) and a three-finger gripper (Gripper Series KG3) were used to perform the grasping and placement tasks. The gripper was mounted at the end of the robotic arm. The robot and its work environment are illustrated in Figure 1C. KINOVA7 is a lightweight robotic arm with seven degrees of freedom, capable of high-precision and high-speed motion control in three-dimensional space, enabling it to perform complex and delicate tasks. The KG3 gripper is an underactuated, multifunctional three-finger gripper that can handle objects of various sizes and shapes. A depth camera (Realsense D455) was fixed to the wrist of the gripper to capture real-time images from the end of the robotic arm, providing a clear control view that was transmitted to the remote control screen. This enables the user to receive real-time updates from the perspective of the gripper.

### 2.2 Experimental procedure

To validate the effectiveness of the brainwave-eye movement-controlled teleoperation robot system, eight right-handed subjects were recruited for the experiment. All of them were healthy and had no known mental or physical diseases. They were all right-handed. Among them, 5 were male and the average age was 26 years old. The subjects used the left and right hand MI paradigms as control signals, while eye movement fixation was used as a mode-switching method to control the robotic arm to perform grasping tasks. To validate the effectiveness of the system, the subjects were required to complete the grasping and placement of objects at three fixed positions in a specified sequence. During the whole experiment, the subjects maintained a stable sitting posture, with their chin placed on a fixed support and both hands relaxed on the desk, and kept their wrists away from the table to avoid contact with the actuators. This study adhered to the principles of the Helsinki Declaration and was approved by the Ethics Committee of the First Affiliated Hospital of Nanjing Medical University (2020-SR-362.A1).

**FIGURE 3:**
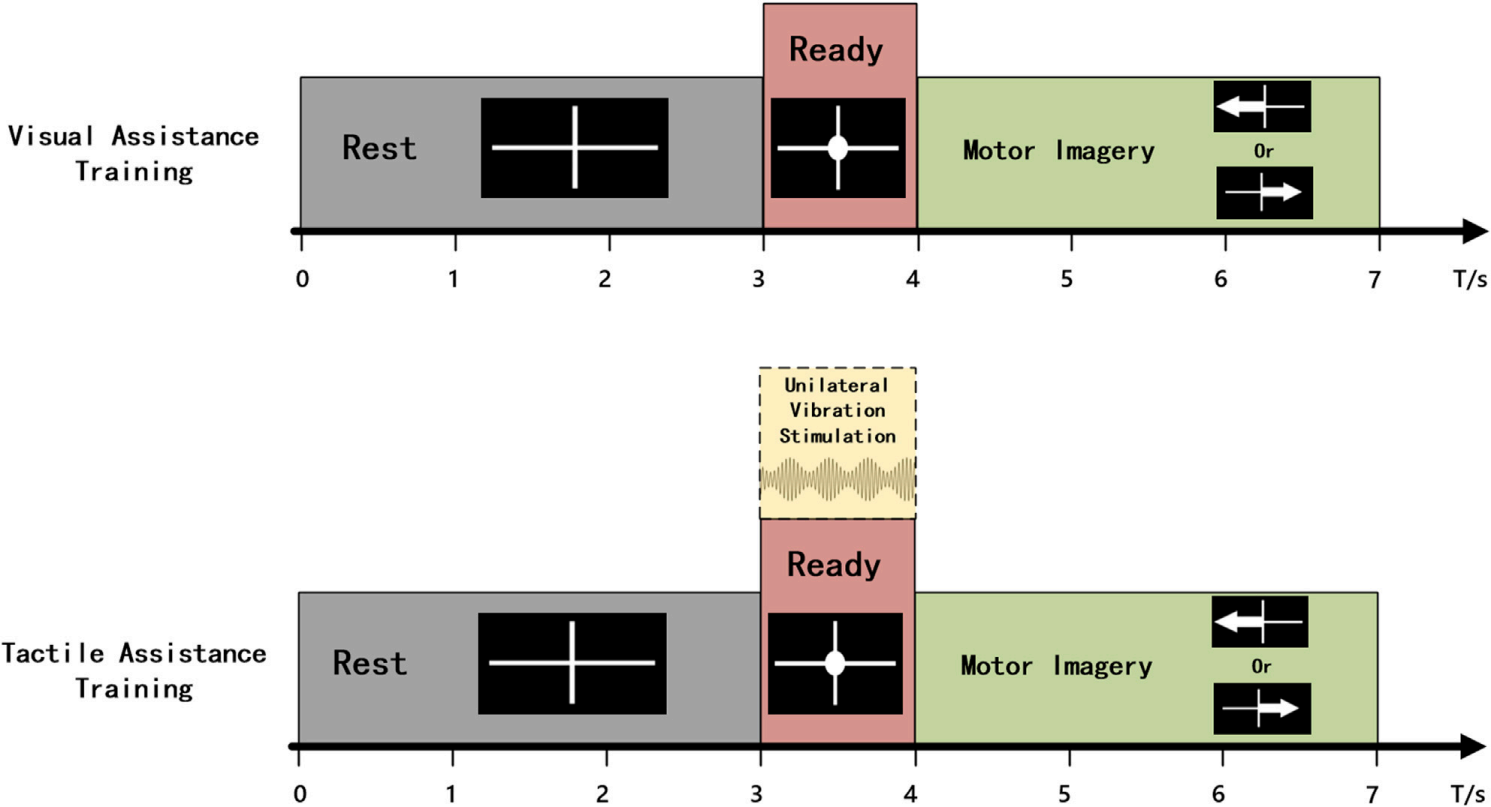
Schematic diagram of the time structure for a single trial of MI training.

Before the control experiment began, the subjects completed an MI training task to train the online classifier. They sequentially performed training for the visual-assistance (VA) and tactile-assistance (TA) groups to avoid the carryover effects of tactile stimulation from influencing the experimental results. Each group consisted of 20 trials of MI for both the left and right hand. The time structure of a single trial is illustrated in Figure 3. In the VA group, the single trial flow was as follows: The screen displayed a white cross for 3 s, indicating that the subject could relax. During the third to fourth seconds, a white circle appeared in the center of the white cross, prompting the subject to prepare for the MI task. A white arrow pointing left or right was then displayed for 3 s, prompting the subject to perform the left- or right-hand MI task. The TA group followed the same time structure as the VA group, with the distinction that from the third second, 1 s vibration stimulation was applied to the median nerve of the wrist of the hand about to perform the imagery task. EEG data from 4.5 to 6.5 s of each trial were used to train the online classifier, with the Common Spatial Pattern (CSP) algorithm for feature extraction and Linear Discriminant Analysis (LDA) for classification. The classifiers trained using both groups of data were used for online classification in the VA control and TA control experiments.

**FIGURE 4:**
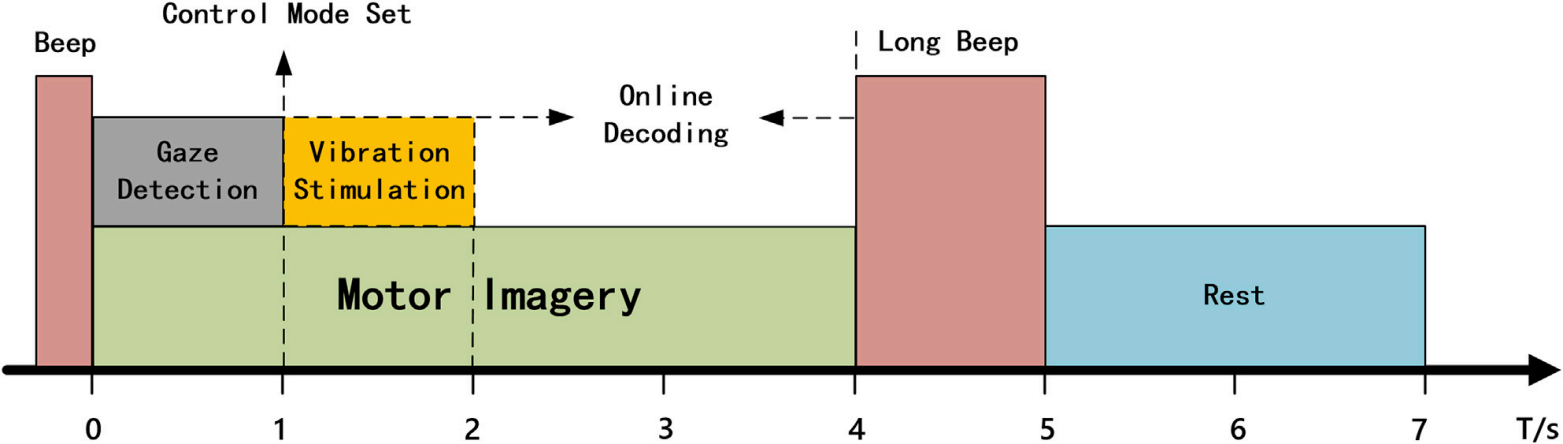
The time structure for a single trial of robot control.

The control experiment was divided into VA and TA two groups, based on the presence or absence of vibration stimulation. In the VA group, the subjects controlled the robotic arm’s movement by imagining the motion of the left or right hand, using the LDA classifier trained with visual assistance to decode the imagined data in real time. In the initial state, the control mode of the robotic arm was as follows: if the subject imagined the left hand movement, the robotic arm moved 5 cm to the left and 45° horizontally; if the subject imagined the right hand movement, the robotic arm moved 5 cm to the right and 45° horizontally. The time flow for a single control task is illustrated in Figure 4. First, the subject hears a beep sound lasting 0.3 s, signaling the start of the task. The subject then focuses their attention, begins to gaze at the target object, and imagines the hand movement. The gaze coordinates during the first second after the beep sound were collected, and the average of these coordinates was calculated to determine the main gaze point for that second. The position of the coordinates was checked to determine whether they fell within one of the three command regions. If so, the corresponding mode switch is executed based on the userâ€™s gaze. If the gaze is in the “Forward” or “Backwards” region, the left or right hand MI command controls the robotic arm to move forward or backward, starting from the next command. If the gaze is in the “Finish” region, the current control task is terminated, and the robotic arm automatically returns to its initial position. To avoid misjudging the imagery time, subjects were required to continuously imagine within 4 s after the beep sound, but only the EEG data from the second to the fourth second were used for online classification. The classification results were converted into control commands and transmitted to the remote robot. A long beep lasting 1 s was heard at the fourth second, signaling the subject to rest. After a 3-s rest, the next control task began.

The difference in the TA group compared to the VA group during the robotic arm control was that whenever the gaze coordinates collected in the first second fall within the yellow or green shaded area shown in Figure 1b, a 1 s vibration stimulation was applied to the left or right wrist. If the gaze was in the blue area, vibration stimulation was applied to both wrists for 1 s to prompt the subject to remain focused. The remaining procedure was identical to that in the VA group.

After each command was issued to the robotic arm, the robot calculated the expected arrival coordinates based on the current end-effector position. If the target position exceeds the control range or enters a nontarget grasping zone, a limit is triggered, and the robotic arm will not execute the movement. If the expected arrival position falls within the designated grasping range of the target, the robotic arm automatically performs position adjustment control after completing the movement, moving the gripper so that the target is centered on the gripper. The distance $D_{i}$ between the end-effector position of the robotic arm and target point was calculated as Formulate 1:

$$D_{i}=\sqrt{{\left(x_{R}-x_{i}\right)}^{2}+{\left(y_{R}-y_{i}\right)}^{2}}$$ (1)

where $x_{R}$ , $y_{R}$ are the coordinates of the end-effector position, and $x_{i}$ , $y_{i}$ are the coordinates of the i -th target.

Once the robotic arm completes the grasping posture adjustment, it enters the grasping and placement control phase. The subject controlled the stepping of the gripper by gazing at the grasping target (the red shaded area at the center of the screen, Figure 1b). Each second of gaze triggers tightening of the gripper. The degree of tightening was determined by the subject’s current attention level, which was quantified by the ratio of the alpha to beta rhythm power from the Cz channel of the EEG. If the subject determines that the object has been successfully grasped, they gaze at the “Finish” area. If the system detects that the subject has gazed at the “Finish” area for more than 1 s, the grasping is considered successful and the target object is automatically grasped and moved to the placement area. After completing the placement, the robotic arm automatically returned to the initial position and waited for the subject to press a button to initiate the next grasping control. The difference between the vibration group and the visual group is that, for each gripper tightening, a 0.4 s vibration stimulus is applied to both wrists of the subject. Prior to the formal grasping experiment, each subject practiced controlling the robotic arm’s movement three to five times to familiarize themselves with the experimental procedure, thus minimizing the impact of skill differences on the experimental results.

### 2.3 Data analysis methods

To analyze the effects of vibration stimulation on MI training and the control system, we performed an offline analysis of EEG signal features under different task conditions. The differences in control efficiency were compared based on the time spent and grasping success rate in various control tasks. Finally, the attention level of the subjects was assessed based on the ratio of alpha and beta rhythm energies in the Cz channel during the grasping task.

In the offline EEG analysis, the SOBI algorithm-based automatic artifact removal (AAR) toolbox (Gómez-Herrero, 2007) was applied to eliminate artifacts such as eye blinks. The signal was then band-pass filtered from to 5–40 Hz to exclude non-relevant frequency bands and re-referenced using the common average reference (CAR) algorithm. Spatial filtering of the imagined signals was performed using the CSP algorithm, followed by the training of an LDA classifier. The between-class scatter matrix and Euclidean distance of the central points for each pair of classes were calculated using the Formulates 2, 3:

$${\tilde{m}}_{i}=\frac{1}{n_{i}}\underset{y\in D_{i}}{\sum }y$$ (2)

$${\tilde{S}}_{B}=\left|{\tilde{m}}_{1}-{\tilde{m}}_{2}\right|$$ (3)

In addition to the feature distribution, we also compared the time-frequency characteristics of the training task. The event-related spectral perturbation (ERSP) was calculated using short-time Fourier transform (STFT) with a Hanning window of 200 ms. The formula used is as follows:

$$\mathrm{ERSP}\left(f,t\right)=\frac{1}{n}\overset{n}{\underset{k=1}{\sum }}\left(F_{k}{\left(f,t\right)}^{2}\right)$$ (4)

where n is the trial number and $F_{k}\left(f,t\right)$ represents the spectral estimate at frequency f and time t for the k -th trial. Data from the second to third seconds of each trial were used as reference data. To analyze the spatial distribution of brain activation during the imagination task, the ERSP values of all electrode channels within the selected frequency bands during 4.5–6.5 s of each trial were averaged and used to generate ERSP topography distribution. To observe the temporal dynamics of brain activation during the imagination task, the C3 and C4 channels, representing the left and right sensorimotor cortices, were used to calculate the ERD from the first to the seventh second of each trial, with the 1.5–2.5 s data selected as the baseline.

In the teleoperation control task, subjects were allowed up to three attempts to grasp each target. If the target was not successfully grasped after three attempts, the task was considered a failure. If the duration of a single attempt exceeded 150 s, or if the subject voluntarily abandoned the attempt, it was considered a failure. The time taken by each subject to move the robotic arm from the initial position to the target grasping range and the time spent adjusting the arm posture after successful grasping were recorded to compare the control efficiency differences between the different control groups.

The ratio of the fast and slow rhythm energies in the EEG reflects the attention level to some extent. In this study, the attention level of the user was assessed by measuring the ratio of the alpha and beta rhythm powers in the Cz channel. A fast Fourier transform (FFT) was performed on 5 s of data after the first grasp attempt to calculate the power spectral density of that segment, from which the average power in the 8–12 Hz and 13–30 Hz frequency bands was extracted to compute the alpha-to-beta ratio. The alpha and beta ratios were averaged across all successful grasping trials for the three target tasks for each subject; this value was used as the subject’s average attention level during the grasping task.

## 3 Results

### 3.1 MI training

In this study, a pre-trained LDA classifier was used to decode MI control commands online, and the classifier was more effective when trained with features that exhibited better separability. Table 1 lists the LDA classifier results from the VA and TA training tasks for all subjects, showing the Euclidean distances between the centers of the feature mappings for the left and righthand MI tasks. This metric reflects the inter-class separability of the two feature sets. The larger the inter-class distance and the smaller the intra-class distance, the better the separability of the two features. From the table, it is evident that the inter-class distance in the TA training group was significantly larger than that in the VA training group (p = 0.017). In six out of the eight subjects, the inter-class distance increased, with many subjects showing more than a twofold increase in distance. Even the two subjects who showed a reduction in distance did not exhibit significant weakening.

**TABLE 1:** The LDA inter class spacing of all subjects.

Group	S1	S2	S3	S4	S5	S6	S7	S8	Ave ± Std
VA	0.739	0.481	0.457	0.446	0.657	0.547	0.525	0.457	0.539 ± 0.10
TA	1.399	0.352	0.468	1.089	1.279	1.144	0.956	0.324	0.874 ± 0.41

**FIGURE 5:**
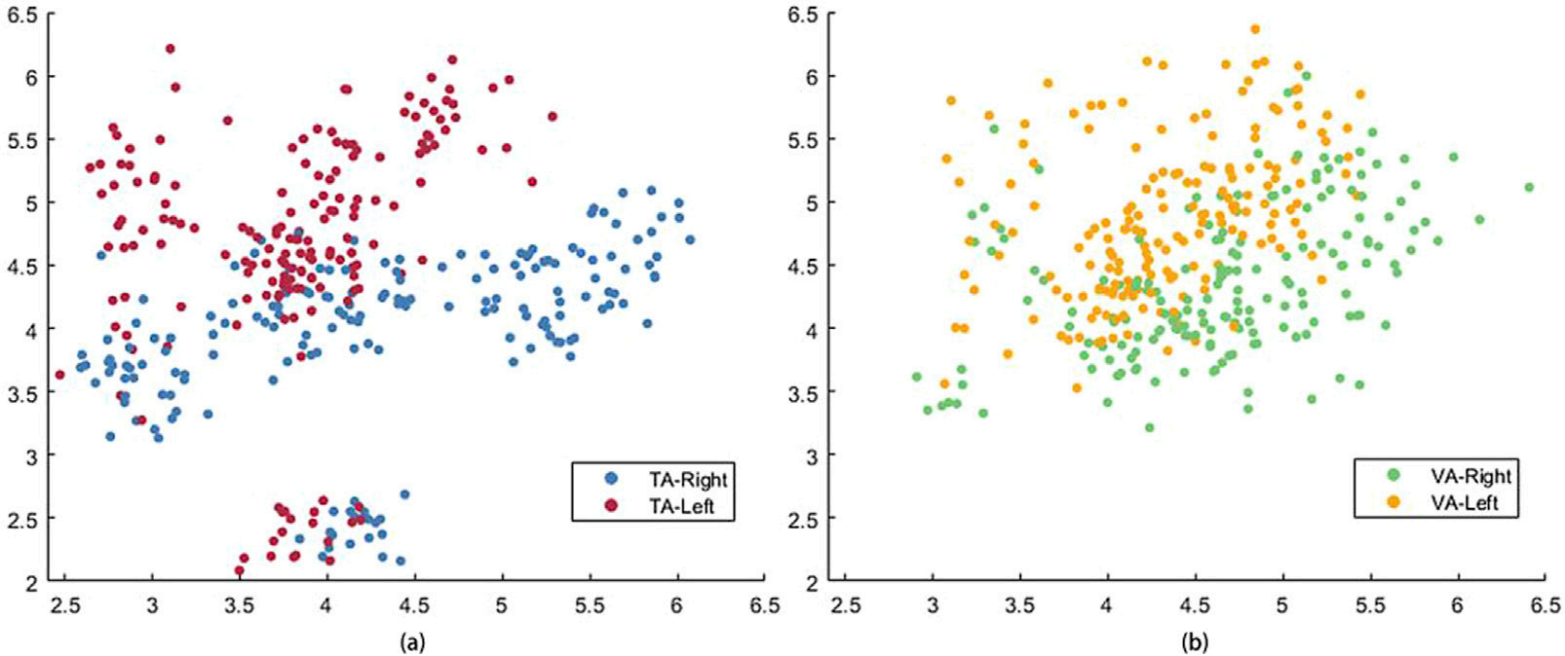
The feature distribution of all subjects in TA **(a)** and VA **(b)** training tasks.

Figure 5 shows the feature distribution across the two training groups for all the subjects. The red and blue circles correspond to the features of the TA training group, and the yellow and green circles correspond to the features of the VA training group. It is clear from the figure that the two classes of features in the VA group are somewhat entangled, leading to errors in classification. In contrast, the TA training group showed greater inter-class separability, with more tightly clustered intra-class distributions, leading to better classification performance.

**FIGURE 6:**
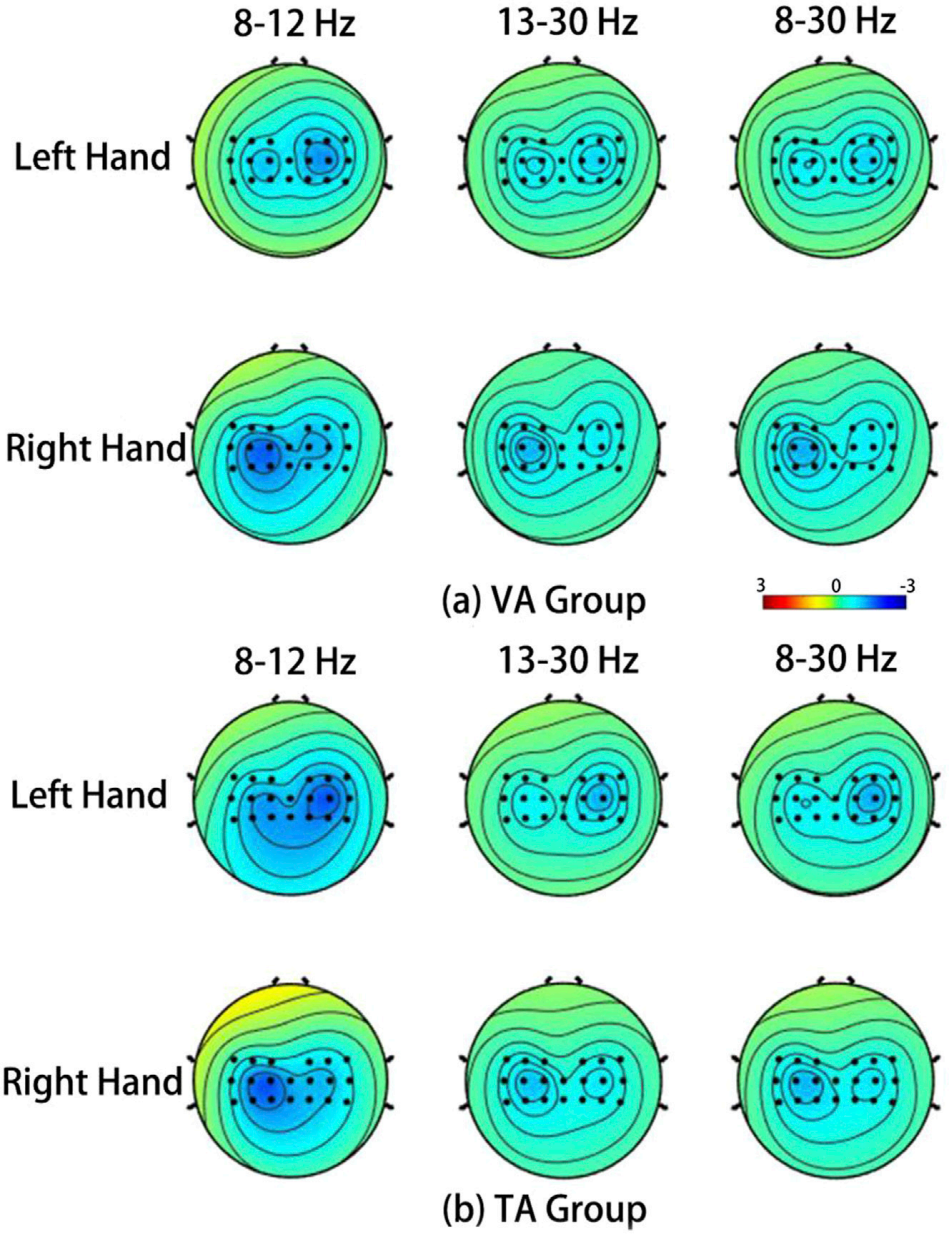
The average ERSP spatial distribution in VA training **(a)** and TA training **(b)** of all subjects during MI.

Figure 6 shows the average ERSP topographies during the MI tasks for all subjects in the VA and TA training groups. The brain activation patterns for the left and right hand MI tasks, as presented in the topographies, indicate that the addition of vibration feedback did not significantly change the activation pattern for the right-hand MI, which remained similar to that of the VA group. However, for left-hand MI, activation in the contralateral sensorimotor cortex was significantly enhanced in the TA group, exhibiting a more pronounced contralateral lateralization. Consequently, the separability of the activation patterns between the two tasks was greater in the TA training group. In the VA group, the left-hand MI produced a relatively weak ERD phenomenon on both sides, but the differences were not significant. In the TA group, the contralateral differences were significantly increased, and the level of difference was more balanced for the right-hand task.

**FIGURE 7:**
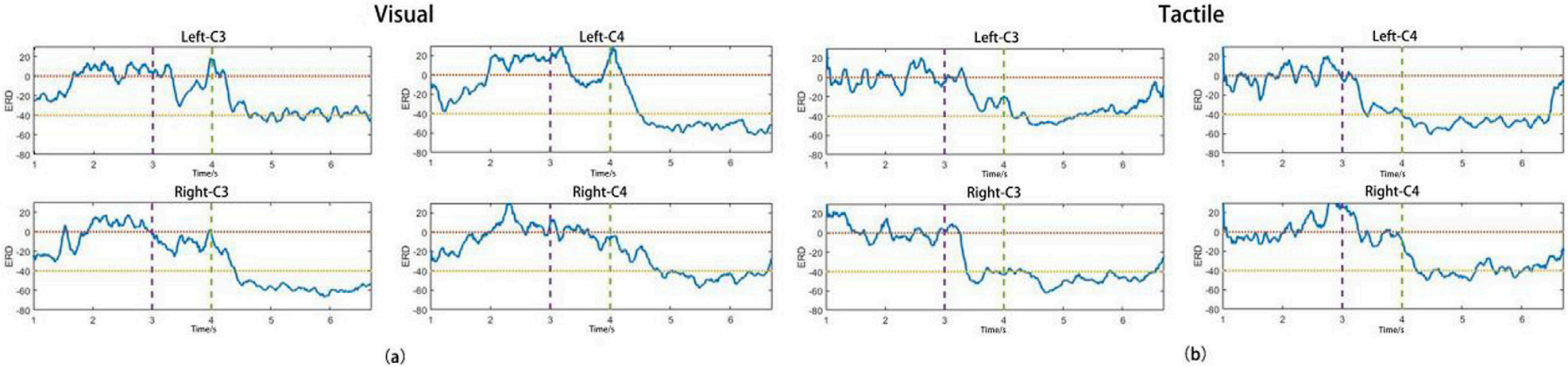
The average ERD curve of all subjects in VA **(a)** and TA **(b)** training.

Figures 7a,b show the average ERD curves for all subjects in the VA and TA training groups, respectively, illustrating the ERD changes during the left and right hand MI tasks at the C3 and C4 channels. The purple dashed lines correspond to the time points when the “prepare” image or vibration stimulation began, and the green dashed lines indicate the start of the MI. The horizontal yellow and orange dashed lines indicate ERD levels of −40% and 0%, respectively. From the figures, we can observe that the two training groups did not show significant differences in the ERD levels during the MI task. The contralateral ERD reached approximately −50% in both groups, suggesting that the addition of vibration stimulation did not significantly enhance brain activation levels during subsequent MI tasks. During the 3–4 s preparation period, the VA group only produced a weak ERD on both sides, which nearly returned to baseline levels before the imagery began. However, in the TA group, the introduction of unilateral vibration stimulation caused the subjects to focus earlier on the side of imagery, and the stimulation also activated the sensory-motor areas on both sides. As a result, the vibration group reached higher ERD activation levels during the preparation phase, which allowed them to reach the ERD peak more quickly than the VA group after imagery began, especially in the contralateral brain regions, with a difference of approximately 0.5 s. Thus, the TA group showed a higher ERD duration and average ERD during the imagery period than the VA group.

### 3.2 Robot control

Table 2 presents the time spent by all subjects to grasp different targets and the number of times tasks were abandoned/failed during the control experiment. The target grasp time shown in the table refers to the fastest time from the start of control to the successful grasp of the target in a single attempt. Failures indicated that the subject failed to grasp the target in all three attempts. Abandonment counts refer to the number of times a task was abandoned because it exceeded 150 s or was voluntarily abandoned by the subject. Because each target allowed only three attempts, the maximum number of abandonments was nine. From the table, it is evident that the failure and abandonment rates in the VA group were significantly higher than those in the TA group, especially for left-sided targets. In the VA group, four subjects failed to complete the grasp, whereas only one subject failed in the TA group.

**TABLE 2:** The time spent by the subject performing robot control.

Subject	Target A/s		Target B/s		Target C/s		Fail times	
	VA	TA	VA	TA	VA	TA	VA	TA
S1	Fail	46.1	49.5	48.3	86.5	49.6	3	0
S2	Fail	45.3	47.8	53.6	97.1	74.3	4	2
S3	55.2	Fail	48.5	Fail	56.5	65.4	4	7
S4	46.1	45.7	Fail	Fail	Fail	57.3	6	4
S5	45.8	45.5	48.2	47.8	Fail	48.4	3	1
S6	Fail	46.7	Fail	46.6	72.8	64.2	8	2
S7	46.8	48.6	51.2	49.1	50.9	68.1	1	2
S8	Fail	47.3	47.5	49.7	57.7	50.5	3	0

Table 3 presents the average ratio of alpha to beta rhythm power at the Cz channel for all subjects during the execution of all grasping tasks. The addition of vibration tactile stimulation significantly improved subjects’ motor attention levels (p = 0.047), with only one subject showing an increase in the TA group. The Cz channel is located in the central sensory-motor area, and most subjects exhibited a decrease in both alpha and beta rhythm energy when concentrating on the grasping tasks. In the TA group, most subjects showed a greater reduction in alpha than beta rhythm, leading to a decrease in the alpha-to-beta power ratio, reflecting an increase in attention levels.

**TABLE 3:** The average attention level of all subjects during the grasping.

Group	S1	S2	S3	S4	S5	S6	S7	S8	Ave ± Std
VA	1.76	5.26	3.67	1.56	3.62	1.89	4.14	3.25	3.14 ± 1.22
TA	1.53	3.22	2.69	2.57	3.15	1.45	2.75	2.89	2.53 ± 0.64

## 4 Discussion

One of the major challenges in brain-controlled robot systems is the use of EEG signals to switch control modes or toggle tasks on and off Han et al. (2020). Among the existing EEG control signals, several types of brain states have been used as brain switches, such as Event-Related Potential (ERP) and SSVEP. However, most related research is still in the feasibility analysis stage, with relatively few brain switches applied to practical control systems. Consequently, researchers have turned to using alternative signals, such as blinks, swallowing, and electromyography (EMG) signals, to complement brain switches. In this study, we used eye-gaze fixation signals as a switch signal to toggle the control modes by determining the location of the gaze on the interface. This switching method benefits from the flexibility of eye gaze fixation, as it does not require the user to make actual movements, making it especially suitable for patients with conditions such as stroke or paralysis that limit their mobility. In this study, the eye-gaze acquisition was accurate, and its robustness was high, with rare occurrences of control process disruptions.

To compare whether the addition of vibration stimulation could improve training efficiency, we limited the number of MI training trials, resulting in insufficient data. This also resulted in a higher misclassification rate during online control. However, the addition of vibration stimulation significantly increased the inter-class separability of the training features, which in turn improved the MI decoding accuracy in the online control. This finding is crucial for improving control efficiency. Previous research has indicated that multimodal feedback, especially visual and tactile feedback, is beneficial for MI training and decoding. For example, Wrist vibration stimulation can enhance activation in the contralateral sensorimotor cortex and improve the decoding accuracy of MI for the non-dominant or hemiparetic hand Shu et al. (2018). Studies have combined hand motion animations and electrical stimulation with MI training to improve sensorimotor cortex activation and classification performance during the MI (Wang et al., 2019). However, such studies typically apply stimulation during the MI task itself, and some studies have replaced visual feedback with tactile feedback as MI task guidance, but these approaches did not show a significant effect on MI performance (Hehenberger et al., 2021).

The application of vibration stimulation before starting MI to assist in focusing on the side of the upcoming imagery differs fundamentally from previous studies, where vibration stimulation was applied during the MI task Osborn et al. (2020); Batistić et al. (2023). Similarly, vibration assistance did not significantly enhance the activation level during the subsequent MI task, particularly in the right-hand MI. However, the enhancement of contralateral sensorimotor cortex ERD in the left-hand task should be attributed to the vibration stimulation compensating for the imbalance in non-dominant hand MI capabilities. Tactile cues on the non-dominant hand helped users engage more naturally in the imagery, mentally shifting their focus to the left side, and reducing the psychological bias towards the dominant hand. Furthermore, the addition of vibration stimulation allowed the subjects to identify the imagined side without visual attention, bypassing the mental process of converting visual commands into movements. This likely led to a stronger focus on imagery, a faster imagery rate, and improved spatial awareness. Owing to the enhanced MI training with vibration assistance, the control efficiency in the vibration group was significantly higher than that in the visual group. From the control results, it is clear that many subjects could not complete the left-side target grasp, possibly due to the weaker MI ability of the non-dominant hand (Tecchio et al., 2006). The addition of vibration stimulation helped mitigate this psychological bias and balanced the MI abilities of both hands.

**FIGURE 8:**
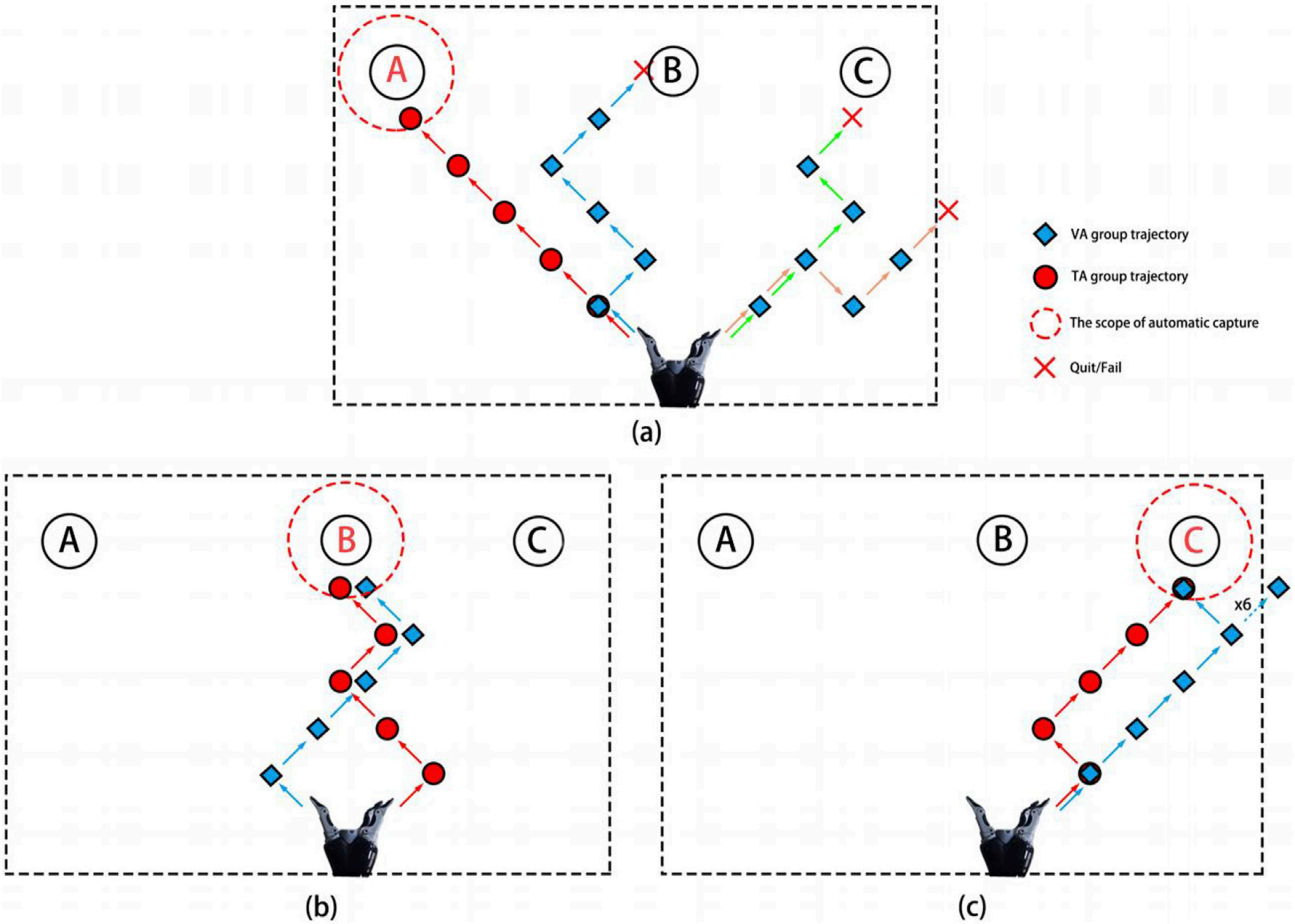
The robot motion trajectories of subject S1 during the process of capturing target A **(a)**, target B **(b)**, and target C **(c)**, respectively.

Figure 8 shows the control trajectory plots for subject S1 when grasping the three targets. The red circles and blue diamonds represent the control trajectories of the TA and VA groups, respectively. The red crosses indicate when the subject abandoned or failed to grasp the target, and the red dashed lines show the automatic grasp range for each target. The subject completed all three target grasps with 100% accuracy in the TA group but successfully grasped only targets B and C in the VA group. From the trajectory plots, it is clear that the decoding rate of the VA group for the left-hand imagery task was low, which prevented them from grasping target A. Additionally, grasping target C required a leftward movement command, but multiple misclassifications led to wasted time as the movement was erroneously recognized as a rightward direction. The subject also reported feeling frustrated and anxious because of these misclassifications, which contributed to his failure to grasp the target object. However, the addition of vibration stimulation helped the subject focus better on the imagined movement, with a stronger spatial direction sense. The increased decoding accuracy also boosted his confidence, enabling him to successfully grasp all three targets. Similarly, many subjects struggled to maintain the same level of imagery performance during the online control process as they did during their training. This may be due to the lack of visual guidance, and multiple misclassifications would cause the robot to deviate significantly from the intended path, leading to greater frustration and difficulty in maintaining stable imagery and creating a negative cycle. In contrast, vibration stimulation helped restore the feedback that the subjects experienced during training, and unilateral stimulation provided better spatial guidance, allowing them to focus more effectively on the imagined side.

Neuroscientific research has shown that attention levels are related to the “neural balance” of brainwave structures, which means that the frequency of brainwaves produced by different brain regions should be similar to each other. Generally, when attention is high, the brain generates more beta waves (13–30 Hz), while low attention is associated with more alpha waves (8–12 Hz) or theta waves (4–7 Hz). Attention can be categorized into two types: top-down and bottom-up (Posner et al., 1984; Leonards et al., 2000). Top-down attention refers to the active concentration on a specific target or task, such as reading, writing, and problem solving. Bottom-up attention refers to passive reactions to external stimuli, such as hearing a sudden sound or seeing a flashing light. Different types of attention involve distinct brain regions. The frontal lobe is responsible for higher cognitive functions and active control, and is thus associated with top-down attention, whereas the parietal lobe handles sensory processing and spatial orientation, and is thus associated with bottom-up attention. In this study, the Cz channel, located in the sensorimotor cortex of the parietal lobe, showed that the addition of vibration stimulation during each grasping task caused suppression of alpha and beta rhythms in the sensorimotor cortex. The experimental results showed that the alpha-to-beta ratio in the TA group was significantly lower than that in the VA group, which may be because the tactile input provided a more intuitive control experience for the subject. The enhanced tactile feedback combined with the control effects formed a sensory-motor feedback loop, subjectively increasing the userâ€™s focus on the task. Therefore, the input of vibration stimulation increases bottom-up attention, further improving the userâ€™s attention level.

Finally, this study exclusively involved healthy subjects to validate the feasibility of the system and the effectiveness of tactile stimulation enhancement. In the future, this system and its tactile augmentation technology hold promise for applications in rehabilitation therapies for patients with brain injuries. Most brain injury conditions may impair functional connectivity in sensory- and motor-related brain regions, and active rehabilitation training may contribute to improvements in functional connectivity within these regions Arun et al. (2020). Previous studies have demonstrated that combining multisensory stimulation with active rehabilitation training can induce more pronounced neural activation in the brain Du et al. (2022). The strategic integration of tactile stimulation into brain-computer interface systems, such as rehabilitation robot, may advance BCI technology across multiple dimensions, including therapeutic efficacy, user acceptance, and system robustness.

## 5 Conclusion

This study designed a teleoperation robotic system based on hybrid EEG and eye-movement signal control, which combined robot position-assisted control. The user is responsible for controlling primary operations, such as target selection and gripper manipulation, whereas the robot autonomously performs detailed operations, such as adjusting the grasp posture and automatic placement. A controlled experiment was conducted to verify the effectiveness of vibration stimulation as an auxiliary feedback method for improving the overall system control efficiency. The results showed that vibration stimulation can effectively assist in MI training, thereby improving the user’s control efficiency of the robot. It also increased the user’s attention during grasping tasks. This system holds great potential for future applications in motor rehabilitation scenarios and to help patients improve their motor and cognitive abilities.

## Data Availability

The raw data supporting the conclusions of this article will be made available by the authors, without undue reservation.
